# Royal Jelly Reduces Cholesterol Levels, Ameliorates Aβ Pathology and Enhances Neuronal Metabolic Activities in a Rabbit Model of Alzheimer’s Disease

**DOI:** 10.3389/fnagi.2018.00050

**Published:** 2018-03-05

**Authors:** Yongming Pan, Jianqin Xu, Cheng Chen, Fangming Chen, Ping Jin, Keyan Zhu, Chenyue W. Hu, Mengmeng You, Minli Chen, Fuliang Hu

**Affiliations:** ^1^College of Animal Sciences, Zhejiang University, Hangzhou, China; ^2^Comparative Medical Research Center, Experimental Animal Research Center, Zhejiang Chinese Medical University, Hangzhou, China; ^3^The First Affiliated Hospital, Zhejiang Chinese Medical University, Hangzhou, China; ^4^Department of Bioengineering, Rice University, Houston, TX, United States

**Keywords:** royal jelly, Alzheimer’s disease, hypercholesterolemia, amyloid plaques, neuronal metabolism activity

## Abstract

Alzheimer’s disease (AD) is the most common form of dementia characterized by aggregation of amyloid β (Aβ) and neuronal loss. One of the risk factors for AD is high cholesterol levels, which are known to promote Aβ deposition. Previous studies have shown that royal jelly (RJ), a product of worker bees, has potential neuroprotective effects and can attenuate Aβ toxicity. However, little is known about how RJ regulates Aβ formation and its effects on cholesterol levels and neuronal metabolic activities. Here, we investigated whether RJ can reduce cholesterol levels, regulate Aβ levels and enhance neuronal metabolic activities in an AD rabbit model induced by 2% cholesterol diet plus copper drinking water. Our results suggest that RJ significantly reduced the levels of plasma total cholesterol (TC) and low density lipoprotein-cholesterol (LDL-C), and decreased the level of Aβ in rabbit brains. RJ was also shown to markedly ameliorate amyloid deposition in AD rabbits from Aβ immunohistochemistry and thioflavin-T staining. Furthermore, our study suggests that RJ can reduce the expression levels of β-site APP cleaving enzyme-1 (BACE1) and receptor for advanced glycation end products (RAGE), and increase the expression levels of low density lipoprotein receptor-related protein 1 (LRP-1) and insulin degrading enzyme (IDE). In addition, we found that RJ remarkably increased the number of neurons, enhanced antioxidant capacities, inhibited activated-capase-3 protein expression, and enhanced neuronal metabolic activities by increasing N-acetyl aspartate (NAA) and glutamate and by reducing choline and myo-inositol in AD rabbits. Taken together, our data demonstrated that RJ could reduce cholesterol levels, regulate Aβ levels and enhance neuronal metabolic activities in AD rabbits, providing preclinical evidence that RJ treatment has the potential to protect neurons and prevent AD.

## Introduction

Alzheimer’s disease (AD), the most common type of dementia, is a primary degenerative disease that occurs in the central nervous system (CNS). About 44 million people are currently suffering from AD worldwide, and this population is estimated to exceed 131 million by 2050 (Comas Herrera et al., 2016). Except for 5% of AD cases that are familial, most of the cases are sporadic AD (SAD), the onset of which is largely influenced by both environmental and genetic factors (Selkoe, 2001; Lacher et al., 2018). Though no cure has been found for AD, medications such as cholinesterase inhibitors and N-methyl D-aspartate (NMDA) receptor antagonists are widely used to treat its symptoms. However, the long-term use of these drugs are known to cause serious side-effects (Fereshtehnejad et al., 2014), therefore mandating the development of alternative treatment options.

Since AD is characterized by amyloid β (Aβ) deposition and neuronal loss in the brain (Li et al., 2015), a growing number of studies have been trying to control AD by inhibiting the formation and deposition of Aβ. It was recently found that active ingredients of certain functional foods have anti-aging effects, indicating that dietary intervention may have the potential to prevent or delay the onset of AD (Celik and Sanlier, 2017). One functional food of particular interest is royal jelly (RJ; Ramadan and Al-Ghamdi, 2012), a secretion produced from the hypopharyngeal and mandibular glands of worker bees for feeding to and developing queen bees. Consumed worldwide as a functional food, RJ consists of proteins, carbohydrates, lipids, free amino acids, vitamins (Takenaka, 1982; Nagai and Inoue, 2004; Pourmoradian et al., 2012), as well as a variety of bioactive compounds, including peptides (Fontana et al., 2004), adenosine monophosphate (AMP) N1-oxide (Hattori et al., 2010), acetylcholine (Wei et al., 2010) and fatty acids such as 10-hydroxy-2-decenoic acid (10-HAD; Butenandt and Rembold, 1957; Ramadan and Al-Ghamdi, 2012). RJ has been shown in multiple studies to have anti-aging (Honda et al., 2011), anti-oxidative (Nagai et al., 2006), lipid-lowering (Vittek, 1995) and anti-inflammatory (Kohno et al., 2004) effects. Furthermore, it was recently reported that RJ significantly improves spatial learning and memory in rats with streptozotocin-induced SAD (Zamani et al., 2012), and that RJ attenuates Aβ toxicity in a *C. elegans* model of AD (Wang et al., 2016). Despite these latest research developments in RJ’s potential treatment effects related to AD, the mechanism of how RJ regulates Aβ formation and delays the development of AD remains elusive.

One hypothesis is that RJ regulates the formation of Aβ via reducing cholesterol levels. Hypercholesterolemia, a known risk factor for AD, has been shown to promote Aβ neurotoxicity, Aβ accumulation and local neuronal loss across epidemiological (Kivipelto et al., 2001; Gonzalo-Ruiz et al., 2006), animal (Sparks and Schreurs, 2003) and cellular (Galbete et al., 2000) studies. It is thought that hypercholesterolemia can enhance the activities of γ-secretase and β-secretase, facilitate the metabolism of amyloid precursor protein (APP), aggravate Aβ deposition, promote the formation of senile plaques and then lead to AD (Kuo et al., 2015; Loke et al., 2017). The potential mechanism by which hypercholesterolemia causes Aβ formation was revealed by Jaya Prasanthi et al. (2008) in which they found that the hypercholesterolemia-induced production of Aβ was correlated with an increased level of β-site APP cleaving enzyme 1 (BACE1) and receptor for advanced glycation end products (RAGE), as well as a decreased level of insulin degrading enzyme (IDE) and low density lipoprotein receptor-related protein 1 (LRP-1).

In this study, we first investigate whether RJ has an effect on cholesterol and Aβ levels using an AD rabbit model. Though transgenic rodents have been used as the main animal model for AD (e.g., APPswe/PS1dE9 double transgenic mice), this model is unsuitable for SAD due to a lack of correct APP protein sequence and a lack of cleavage enzymes to trigger Aβ peptide formation (Liu et al., 2014). In contrast, rabbits naturally produce cleavage enzymes for Aβ peptides, and their Aβ peptide sequence is identical to that of humans (Johnstone et al., 1991). Notably, the connection between cholesterol levels and Aβ plaques was first reported in rabbits (Sparks et al., 1994), in which rabbits showed a marked response to high cholesterol diets and exhibited Aβ deposition in plaques. Furthermore, Sparks and Schreurs (2003) found that after adding copper to the diet of cholesterol-fed rabbits, the rabbits developed cortical amyloid deposits and exhibited at least 12 other pathological features that are observed in the brain of human AD patients, such as learning deficits. These findings together highlight the usefulness of the rabbit model, and demonstrate the potential of using rabbits for preclinical drug evaluations (Woodruff-Pak et al., 2007).

To provide potential mechanisms of how RJ regulates Aβ deposition and potentially improves AD in rabbit models, we assess the effects of RJ on a variety of biological factors, including the expression of proteins involved in the production, translocation and clearance of Aβ, anti-oxidative capacities, neuronal loss, and neuronal metabolic activities. In particular, the effects of RJ on neuronal metabolic activities are measured using high-field proton magnetic resonance spectroscopy (^1^H-MRS), which is a non-invasive neuroimaging technique extensively used to quantify metabolic changes in AD pathology (Chen et al., 2012). We use ^1^H-MRS to evaluate the levels of four main metabolites of AD (i.e., N-acetyl aspartate (NAA), choline (Cho), glutamate (Glu) and myo-inositol (mI)), since numerous studies have shown that NAA and glutamate are decreased in AD, whereas Cho and mI are increased in the early stage of AD (Ackl et al., 2005; Zhang et al., 2014).

## Materials and Methods

### Animals

A total of 24 male White Hair and Black Eyes (WHBE) rabbits (3–4 months old, 1.8–2.0 kg) were purchased from Xin Jian rabbit field (Certificate No. SCXK, Zhejiang, 2015-0004, China), Dashiqu town, Xinchang City (Zhejiang, China). They were housed individually under a 12-h light/dark cycle and were provided with food and water *ad libitum*. All experiments were approved by the Institutional Animal Care and Use Committee of Zhejiang Chinese Medical University (IACUC Approval No. ZSLL-2016-115), and were performed according to the guidelines from the Laboratory Animal Research Center of Zhejiang Chinese Medical University (Certificate No. SYXK, Zhejiang, 2013-0184, China).

**Figure 1:**
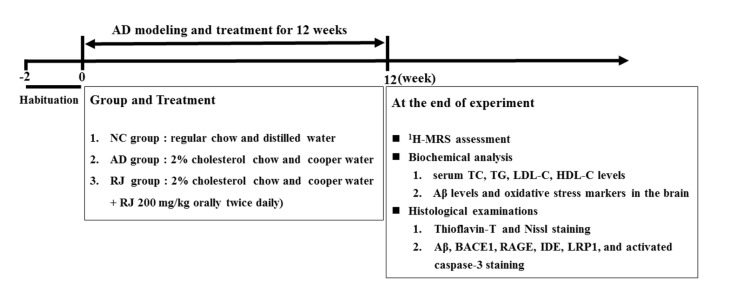
A flowchart of the experimental design in this study.

After adaptation to the environment for 14 days, the rabbits were randomly divided into three groups (*n* = 8 rabbits per group): normal control (NC) group, AD model group and RJ intervention (RJ) group. The NC group rabbits were fed with a regular chow and distilled water (DW), whereas the AD model group was fed with chow plus 2% cholesterol and DW plus cooper (0.12 ppm cooper ion as sulfate) as used by Sparks and Schreurs (2003). The RJ group was fed with chow plus 2% cholesterol and DW plus cooper, and received 200 mg/kg of RJ (Jiangshan, Zhejiang, China) via oral administration twice a day (a total dose of 400 mg/kg RJ daily) in the morning and the afternoon for 12 weeks. This dose was selected based on the previous report that an oral administration of 6 g RJ per day can significantly reduce cholesterol levels in human clinic therapy (Guo et al., 2007). The duration of the experiment procedure was 12 weeks. The experimental designs of biochemical analysis, ^1^H-MRS assessment and histological examinations are shown in Figure 1. After 12 weeks of administration, ^1^H-MRS assessment, a set of biochemical index in the serum and brain, as well as all AD pathological indices were investigated.

### Serum Total Cholesterol (TC), Triglycerides (TG), Low-Density Lipoprotein Cholesterol (LDL-C) and High-Density Lipoprotein Cholesterol (HDL-C) Levels Measurements

After 12-week administration, rabbits (eight per group) would first fast for 12 h, and then blood samples were drawn from their middle auricular artery. Automatic biochemical analyzer (7020, HITACHI, Japan) was used to analyze total cholesterol (TC), triglycerides (TG), low-density lipoprotein cholesterol (LDL-C) and high-density lipoprotein cholesterol (HDL-C) in blood samples by kits corresponding to each component (Shanghai Shenneng-DiaSys Diagnostic Technology Co., Ltd., China).

### Quantification of Aβ Levels by ELISA

For ELISA test of Aβ1–40 and Aβ1–42 levels in the rabbit brain (six cortex and hippocampus per group), the wet mass of 100 mg cortex and the hippocampus tissues were sequentially homogenized in a 8× mass of cold 5 M guanidine hydrochloride/50 mM Tris HCl buffer using an IKA ULTRA-TURRAX homogenizer (IKA^®^-werke Gmbh and Co., KG, German). The homogenates were mixed for 4 h at room temperature. Samples were diluted with Dulbecco phosphate buffered saline with 5% BSA and 0.03% Tween-20 supplemented with 1× protease inhibitor mixture, and were centrifuged at 10,000 rpm for 20 min at 4°C (Jaya Prasanthi et al., 2008). The supernatants were then decanted and stored at −80°C until used. The samples were diluted with at least 1:2 standard dilution buffer, and the ELISA kits (Jiancheng Bioengineering Institute, Nanjing, China) were used to measure Aβ1–40 and Aβ1–42 levels according to the manufacturer’s instructions^1^. The protein concentrations of all samples were determined by standard bicinchoninic acid (BCA) assay (Pierce, Rockford, IL, USA; Baptiste et al., 2004). The Aβ levels were normalized to the total protein content in the samples.

### Determination of Superoxide Dismutase (SOD) Activities, Malonaldehyde (MDA) Contents and Reactive Oxygen Species (ROS)/Reactive Nitrogen Species (RNS) Levels

The superoxide dismutase (SOD) and malonaldehyde (MDA) contents in the brain (eight per group) were measured using colorimetric commercial kit (Jiancheng Bioengineering Institute, Nanjing, China). The SOD activity was examined with xanthine oxidase method, and the MDA content was examined with sulfur barbituric acid method (Wang et al., 2005). In addition, reactive oxygen species (ROS)/reactive nitrogen species (RNS) levels in the brain (six per group) were detected with a commercially available ELISA kit (Yuanye Biotech Co., Ltd., Shanghai, China) following the procedure described by the manufacture. Briefly, a 5% w/v cortex and hippocampus homogenates were made in pre-chilled saline using an IKA ULTRA-TURRAX homogenizer (IKA^®^-werke Gmbh and Co., KG, German). The obtained homogenate was centrifuged at 3000 rpm for 20 min at 4°C. The supernatants were stored at −80°C until used. The protein concentration of all samples was determined by Coomassie brilliant blue method. SOD activities, MDA contents, and ROS/RNS levels were then normalized to the total protein content in the samples.

### ^1^H-MRS Assessment

^1^H-MRS was conducted using a 3.0 T MRI scanner (GE Discovery MR 750, GE, USA) coupled with an 8-channel rabbit dedicated coil (Shanghai Chenguang Medical Technology Co., Ltd., China). All rabbits (six per group) were examined by MRI at 12 weeks. Rabbits were anesthetized by intramuscular injection of 30 mg/kg ketamine and 4 mg/kg xylazine 15 min before imaging. Animals were fixed in prone position in the experiment. During examination, the body temperature was maintained at 36–37°C, and their breathing was kept smooth. T2-weighted fast spin-echo sequence (FSE) was used with the following parameters: TR = 5500 ms, TFE = 100 ms, slice thickness = 3 mm, field of view (FOV) = 100 mm × 100 mm, matrix = 352 × 256, NEX = 2. Images were used to guide MRS subsequent location checks. ^1^H-MRS scanning used point-resolved water suppression plus sequence (PRESS) with the following parameters: TR = 2000 ms, TE = 35 ms, voxel = 9 mm × 9 mm × 9 mm, total number of scans = 128, NEX = 8. The region of interest (ROI) was positioned in the hippocampus and part of frontal cortex in the brain (Figure 7A). Pre-scan, automatic shimming and moisture suppression were performed prior to ^1^H-MRS. The shimming effect was expressed as full width at half maximum (FWHM). If the FWHM was higher than 20 Hz, a manual shimming was used to obtain good magnetic field uniformity. The spectra of all rabbits were analyzed using an LC Model (version 6.3, Provencher SW; Provencher, 1993). The chemical metabolites, including NAA, glutamate, Cho, mI and creatine (Cr) were identified. Since Cr is consistent in various diseases, it was used as an internal standard to calculate the ratio of NAA/Cr, glutamate/Cr, Cho/Cr and mI/Cr.

**Figure 2:**
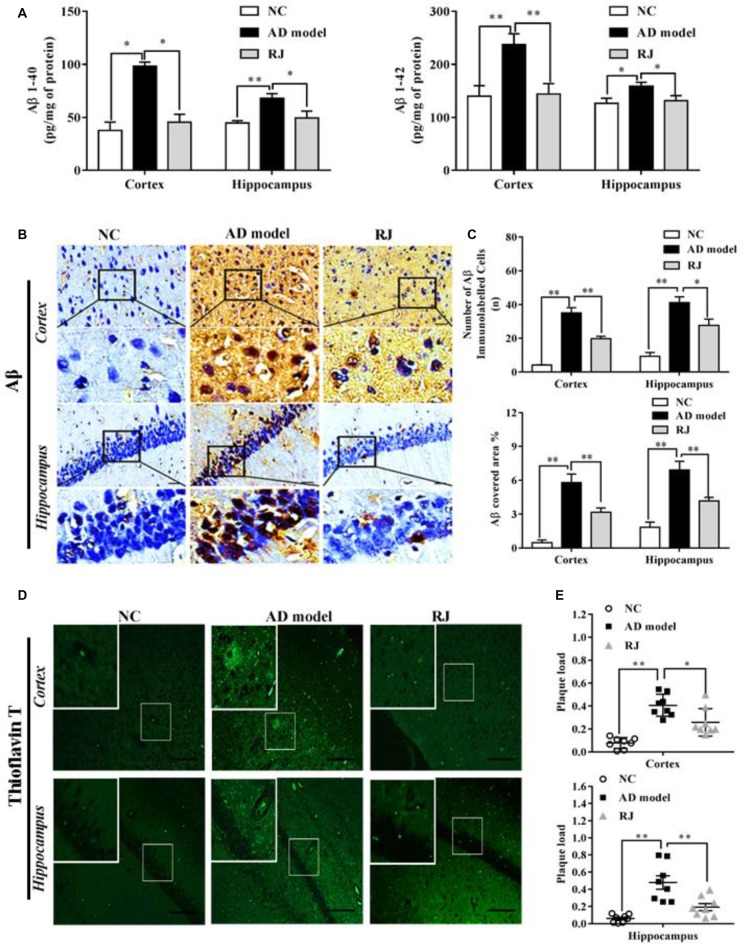
Royal jelly (RJ) decreased brain amyloid β (Aβ) levels and alleviated Aβ pathology in Alzheimer’s disease (AD) rabbits. **(A)** The levels of Aβ1–40 and Aβ1–42 in the cortex and the hippocampus of each group measured by ELISA, *n* = 6 rabbits per group.** (B)** Representative Aβ-staining images in the cortex and the hippocampus of each group. Scale bar = 50 μm. Black squares indicate images with higher-magnification. Aβ positive (brown-colored) was detected mainly in the cytoplasm of neuronal cells and the cytoplasm and membranes of endothelia cells.** (C)** The number of Aβ immunolabeled cells per view (40×) and the covered area of Aβ staining in the brain of each group, *n* = 6–8 rabbits per group. **(D)** An example image of Aβ plaque immunoreactivity in the cortex and the hippocampus of each group by thioflavin-T staining. Scale bar = 50 μm. White squares indicate images with higher-magnification. **(E)** Quantification of thioflavin-T positive deposits in the cortex and the hippocampus of the three groups, *n* = 8 rabbits per group. Data are presented as mean ± SEM. ANOVA with *post hoc* Tukey’s test was used **(A,C,E)**. *P < 0.05, **P < 0.01 vs. the AD model group.

**Figure 3:**
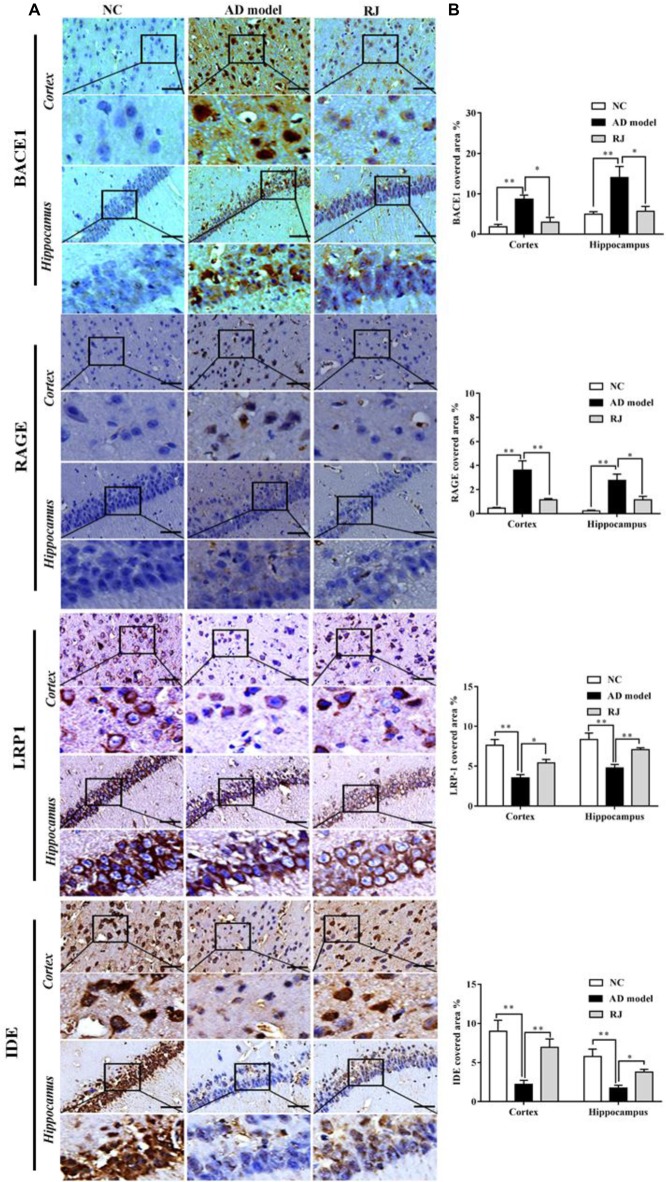
RJ inhibited β-site APP cleaving enzyme-1 (BACE1) and receptor for advanced glycation end products (RAGE) expression levels, and increased lipoprotein receptor-related protein 1 (LRP1) and insulin degrading enzyme (IDE) expression levels, demonstrated by immunohistochemistry. **(A)** Staining for BACE1, RAGE, LRP1 and IDE using specific antibodies in the brain of the AD model, RJ and normal control (NC) group. Scale bar = 50 μm. Black squares indicate images with higher-magnification. **(B)** Quantification of the covered areas of BACE1, RAGE, LRP1 and IDE staining in the frontal cortex and the hippocampus of the three groups, *n* = 6–8 rabbits per group. Data are presented as mean ± SEM. ANOVA with *post hoc* Tukey’s test was used. *P < 0.05, **P < 0.01 vs. the AD model group.

**Figure 4:**
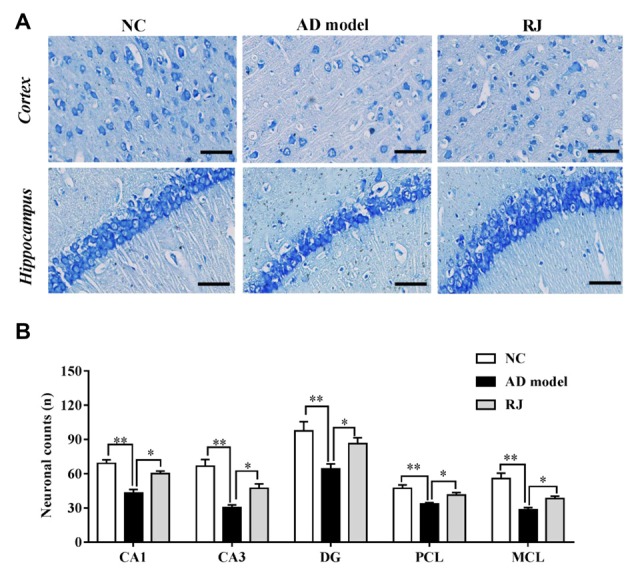
RJ rescued neuronal loss in the brain of AD rabbits. **(A)** Nissl-staining images in the frontal cortex and the hippocampus of the AD model, RJ and NC group. Scale bar = 50 μm. **(B)** Neuronal counts per view (40×) in the hippocampal CA1, CA3, DG regions and cortical pyramidal cellular layer (PCL) and multiform cellular layers (MCL) areas among the three groups, *n* = 6 rabbits per group. Data are presented as mean ± SEM. ANOVA with *post hoc* Tukey’s test was used. *P < 0.05, **P < 0.01 vs. the AD model group.

**Figure 5:**
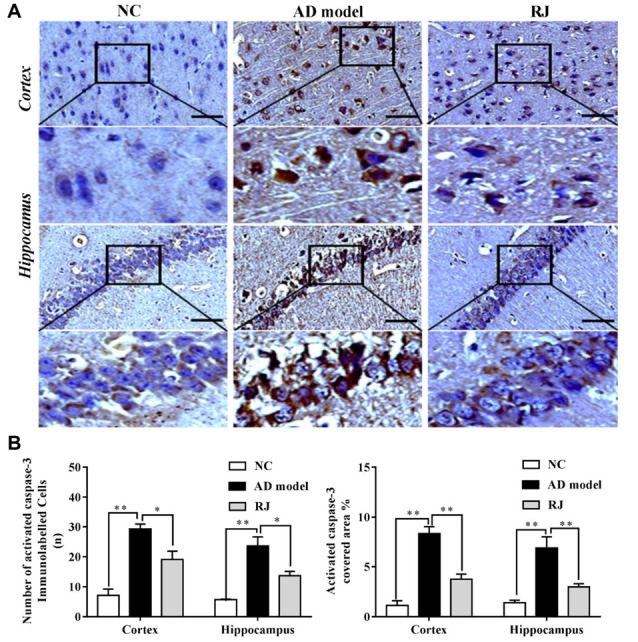
RJ reduced activated caspase-3 expression levels in the brain of AD rabbits. **(A)** Representative activated caspase-3 staining sections in the brain of the AD model, RJ and NC group. Scale bar = 50 μm. Black squares indicate images with higher-magnification. **(B)** The number of activated caspase-3 immunolabeled cells per view (40×) and the covered area of activated caspase-3 staining in the frontal cortex and the hippocampus of the three groups, *n* = 6 rabbits per group. Data are presented as mean ± SEM. ANOVA with *post hoc* Tukey’s test was used. *P < 0.05, **P < 0.01 vs. the AD model group.

**Figure 6:**
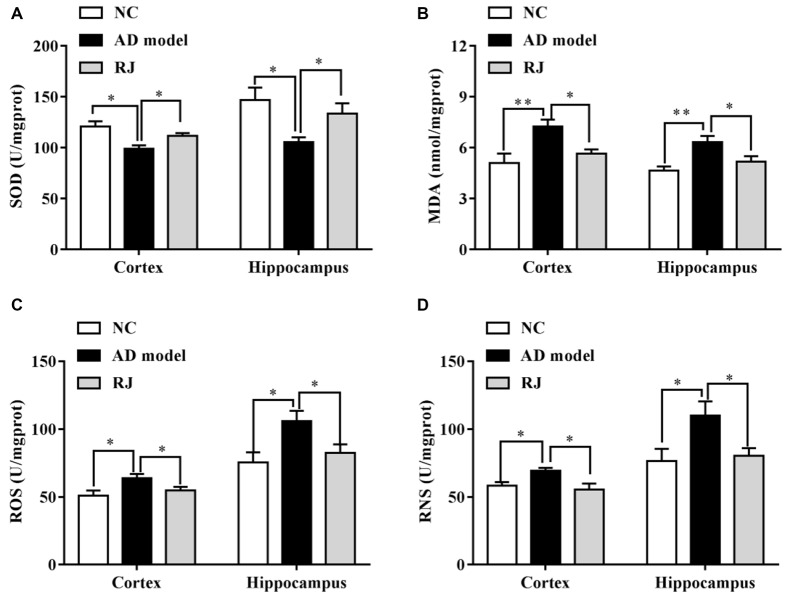
RJ enhanced the anti-oxidative capacities in the brain of AD rabbits. **(A)** Changes of superoxide dismutase (SOD) activities in the cortex and the hippocampus of the three groups, *n* = 8 rabbits per group. **(B)** Changes of malonaldehyde (MDA) contents in the cortex and the hippocampus of the three groups, *n* = 8 rabbits per group. **(C)** Changes of reactive oxygen species (ROS) levels in the cortex and the hippocampus of the three groups, *n* = 6 rabbits per group. **(D)** Changes of reactive nitrogen species (RNS) levels in the cortex and the hippocampus of the three groups, *n* = 6 rabbits per group. Data are presented as mean ± SEM. ANOVA with *post hoc* Tukey’s test was used **(A–D)**. *P < 0.05, **P < 0.01 vs. the AD group.

**Figure 7:**
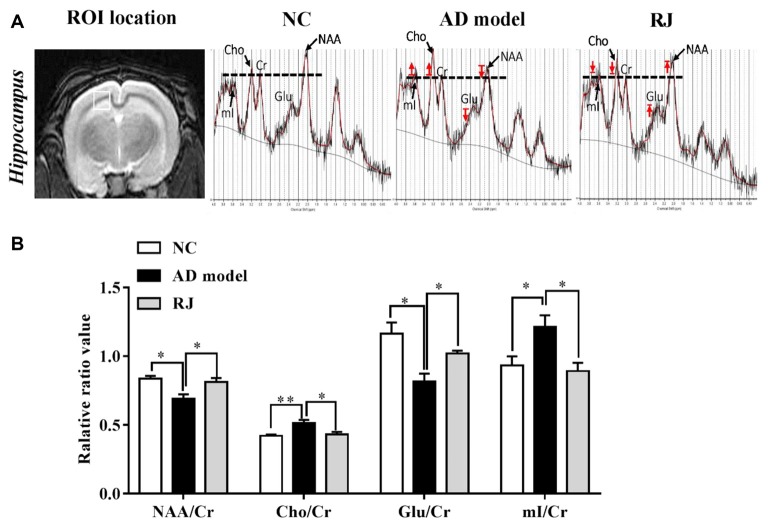
RJ improved brain metabolic activities in AD rabbits. **(A)** Regions of interest (ROI) location in the brain (white box) and representative spectra from the AD model, RJ and NC group rabbits. The red arrow indicates direction of metabolic changes, and the line represents the control level of each metabolite. **(B)** The relative ratios of each metabolite to creatine (Cr) in the brain of the three groups, *n* = 6 rabbits per group. Data are presented as mean ± SEM. ANOVA with *post hoc* Tukey’s test was used. *P < 0.05, **P < 0.01 vs. the AD model group.

### Histological Examinations

All rabbits were euthanized with pentobarbital sodium, and were then perfused with 300 ml 4°C PBS solution after cardiac perfusion. The brain was removed, segmented into 5 mm thick parallel coronal slices, and then fixed in formalin solution for at least 24 h. The selected slices were dehydrated with gradient ethanol, embedded in paraffin and cut into 6 μm coronal sections. They were then stained immunohistochemically with Aβ, beta-site APP cleaving enzyme 1 (BACE1), receptor for advanced glycation end products (RAGE), LDL receptor related protein 1(LRP1), insulin-degrading enzyme (IDE), activated caspase-3, thioflavin-T and Nissl.

1. Thioflavin-T staining was used to evaluate the amount of amyloid-β protein (Zhang et al., 2013), with eight samples in each group. Briefly, slides were deparaffinized and rehydrated in descending grades of ethanol, placed in Mayer hematoxylin for 5 min, rinsed twice in double DW, incubated with 1% thioflavin-T (Dalian Meilun Biological Technology Co. Ltd., China) for 10 min and three changes of DW washes, cover-slipped in a neutral glycerol, and examined with the Hg-Lamp for fluorescence excitation by a Zeiss inverted microscope (Axiovert 200, Carl Zeiss, USA).
2. Nissl staining was used to evaluate neuronal loss (Chen et al., 2009), with six samples in each group. Briefly, slides were hydrated and incubated in 1% toluidine blue solution at 56°C for 30 min, washed with PBS, decolorized with 95% ethanol, dehydrated, dried and cover-slipped.
3. Aβ, BACE1, RAGE, IDE, LRP1 and activated caspase-3 staining, with six to eight samples in each group. Briefly, sections were blocked with 3% hydrogen peroxide solution for 10 min, and were then incubated with primary antibodies overnight at 4°C, including BACE1 (1:100, Santa Cruz Biotechnology, USA), RAGE (1:100, Santa Cruz Biotechnology, USA), IDE (1:100, Santa Cruz Biotechnology, USA), LRP1(1:300, Diage Biological Technology co. Ltd., China), activated caspase-3 (1:200, Biosynthesis Biological Technology co. Ltd., China) and β-amyloid (B-4, 1:100, Santa Cruz Biotechnology, USA, which recognizes APP and Aβ). PBS was used as negative control instead of the primary antibody. Subsequently, the sections were washed with PBS three times and were incubated with secondary antibody for 1 h at room temperature. Sections were visualized with DAB (ZSJQ, Beijing, China) and counterstained with hematoxylin.

All sections were scanned using a Hamamatsu Skeleton Scanner (Nanozoomer S210, Hamamatsu, Japan). Since the main pathological feature of early AD is cortical atrophy, especially in the hippocampus and the medial temporal lobe, we examined the pathological changes in the cerebral cortex and hippocampus. The captured images were examined in a blinded manner by an observer who was unware of experimental condition, and were analyzed using the Image pro plus 6.0 software (Media Cybernetics, Rockville, MD, USA). On Nissl-stained sections, five microscopic fields of the hippocampal CA1, CA3 and DG regions, the cortical cone (pyramidal cellular layer, PCL) and the polymorphic cell layer (multiform cellular layers, MCL) were selected. The neuronal counts in each view were calculated at 40× magnification. We counted Aβ and activated caspase-3 immunolabeled positive cells under 40× magnification in three random fields from the cerebral cortex and hippocampal CA1 areas in the three groups and used the averaged value. For quantification, we calculated Aβ, BACE1, RAGE, IDE, LRP1 or activated caspase-3-postive area under 40× magnification in three random fields from the cerebral cortex and hippocampal CA1 areas, and the staining was quantified as the percent positive staining area as the fraction of immunopositive staining to total area measured. In addition, plaque load was categorized based on the percentage area of tissues positive for deposits labeled by thioflavin-T immunoreactivity.

### Statistical Analysis

Data were expressed as means ± SEM. Statistical analyses were performed using GraphPad Prism 6.0 (GraphPad Software, Inc., La Jolla, CA, USA). All quantitative results were analyzed by one-way ANOVA with *post hoc* Tukey’s test (*p < 0.05, **p < 0.01).

## Results

### RJ Reduced Plasma TC and LDL-C Levels

We first assessed the effects of RJ on blood liquid levels (i.e., TC, HDL-C, LDL-C and TG) in AD rabbits. As shown in Table 1, the levels of TC, HDL-C and LDL-C in the AD model group were higher than those in the NC group (ANOVA with *post hoc* Tukey’s test, all *p* < 0.01). Compared with the AD model group, plasma TC and LDL-C levels in the RJ group were significantly reduced by 28% and 23% respectively (all *p* < 0.05), while there was no significant difference in plasma HDL-C levels between the RJ group and the AD model group (*p* = 0.07). In addition, there was no significant difference in body weight and TG levels among the three groups (ANOVA, *F* = 0.08, *p* = 0.925; *F* = 2.555, *p* = 0.103, respectively).

**Table 1:** Effect of royal jelly (RJ) on weight and blood liquid levels in Alzheimer’s disease (AD) rabbits at 12 weeks.

Parameters	NC group	AD model group	RJ group
Weight (kg)	2.80 ± 0.07	2.83 ± 0.09	2.85 ± 0.07
TC (mmol/L)	0.62 ± 0.09**	45.79 ± 2.97	33.04 ± 4.07*
HDL-C (mmol/L)	0.28 ± 0.05**	2.91 ± 0.10	2.51 ± 0.17
LDL-C (mmol/L)	0.32 ± 0.04**	32.29 ± 1.97	24.86 ± 2.76*
TG (mmol/L)	0.69 ± 0.04	1.91 ± 0.64	0.95 ± 0.25

TC, total cholesterol; HDL-C, high-density lipoprotein cholesterol; LDL-C, low-density lipoprotein cholesterol; TG, triglycerides. Data are presented as mean ± SEM from eight rabbits in each group. ANOVA with *post hoc* Tukey’s test was used. *P < 0.05, **P < 0.01 vs. the AD model group.

### RJ Decreased Aβ Levels and Amyloid Burden in the Cortex and the Hippocampus of AD Rabbits

To observe the effects of RJ on Aβ pathology in the brain of AD rabbits, the Aβ level and amyloid burden were assessed in the three groups of rabbits. As shown in Figure 2A, the Aβ1–40 and Aβ1–42 levels were significantly increased in the cortex area (ANOVA with *post hoc* Tukey’s test, *p* < 0.05 and *p* < 0.01, respectively) and the hippocampus area (ANOVA with *post hoc* Tukey’s test, *p* < 0.01 and *p* < 0.05, respectively) in the AD model group compared with the NC group. In contrast, compared with the AD model group, the RJ group showed a 17%–54% reduction in both Aβ1–40 and Aβ1–42 levels in the cortex area (*p* < 0.05 and *p* < 0.01, respectively) and the hippocampus area (all *p* < 0.05).

Next, the Aβ plaque was evaluated using immunohistochemical analysis (Figure 2B) and thioflavin-T staining. In the frontal cortex and the hippocampus regions, we found that the expression of Aβ-positive proteins was low in the NC group, upregulated in the AD model group, and suppressed in the RJ group. Quantification analysis showed that the number of Aβ immunolabeled cells were significantly reduced in the cortex and the hippocampus of the RJ group compared to the AD model group (ANOVA with *post hoc* Tukey’s test, *p* < 0.01 and *p* < 0.05, respectively). Similarly, the Aβ covered areas were reduced by approximately 45% in the cortex and by 40% in the hippocampus of the RJ group compared to the AD model group (ANOVA with *post hoc* Tukey’s test, all *p* < 0.01; Figure 2C).

Using thioflavin-T staining, a fluorescein that specifically binds to amyloid deposits and can be excited to produce green fluorescence, we found that the RJ group significantly reduced the amount of thioflavin-T positive plaques in both the cortex and the hippocampus areas compared to the AD model group (Figure 2D). Quantitatively, the RJ group reduced the plaques load by 37% in the cortex area (ANOVA with *post hoc* Tukey’s test, *p* < 0.05) and by 60% in the hippocampus area relative to the AD model group (*p* < 0.01; Figure 2E), demonstrating that RJ could greatly decrease Aβ deposition in AD brains.

### RJ Reduced Aβ Accumulation by Decreasing BACE1 and RAGE Expression, and by Increasing IDE and LRP1 Expression

To investigate how RJ reduces Aβ accumulation, the expression of BACE1, RAGE, LRP1 and IDE were measured in the rabbit brains of each group by immunohistochemistry. As shown in Figure 3, compared with the NC group, the expression levels of BACE1 and RAGE were significantly increased in the cortex and the hippocampus of the AD model group (ANOVA with *post hoc* Tukey’s test, all *p* < 0.01). In specific, the BACE1 covered area in the RJ group was significantly decreased by 65% in the cortex and by 59% in the hippocampus compared with the AD model group (all *p* < 0.05). Similarly, the RAGE covered area in the RJ group was markedly reduced by 68% in the cortex (*p* < 0.01) and by 59% in the hippocampus (*p* < 0.05) relative to the AD model rabbits. In contrast, LRP1 and IDE expression levels were markedly decreased in the AD model group (ANOVA with *post hoc* Tukey’s test, all *p* < 0.01). The LRP1 covered area in the RJ group was dramatically increased by 53% in the cortex and by 67% in the hippocampus relative to the AD model group (all *p* < 0.05). Moreover, the IDE covered area in the RJ group was significantly increased by 2.15 folds in the cortex (*p* < 0.01) and by 1.19 folds in the hippocampus relative to the AD model group (*p* < 0.05). Taken together, these results indicate that RJ may reduce Aβ accumulation in AD brains by inhibiting the expression of BACE1 and RAGE as well as by promoting the expression of LRP1 and IDE.

### RJ Reduced Neuronal Loss and Inhibited Apoptosis in AD Rabbit Brains

Nissl staining was performed to evaluate the effects of RJ on neuronal loss in the AD brain. As shown in Figure 4, when compared with the NC group, the AD rabbits showed a typical Alzheimer’s pathology, including nucleus shrinkage, neuronal loss and disappearance of Nissl bodies in the cortex and the hippocampus (Figure 4A). The cell organization was notably improved in the RJ group, in which RJ treatment significantly increased the number of neurons in the hippocampal CA1, CA3, DG regions and cortical PCL and MCL areas by 40%, 56%, 34%, 23% and 34% respectively compared to the AD model group (ANOVA with *post hoc* Tukey’s test, all *p* < 0.05; Figure 4B). These results suggest that RJ treatment can potentially increase the number of neurons and improve neuronal structures in the AD brain.

To confirm the results above, we assessed the expression level of activated caspase-3 in the brain of each group by immunohistochemistry. As shown in Figure 5, the number of activated caspase-3 immunolabeled cells as well as the covered areas of activated caspase-3 were both significantly increased in the cortex and the hippocampus of the AD model group compared to the NC group (ANOVA with *post hoc* Tukey’s test, all *p* < 0.01). In contrast, the number of activated caspase-3 immunolabeled cells were notably decreased in the cortex and the hippocampus of the RJ group compared to the AD model group (all *p* < 0.05). Meanwhile, the covered areas of activated caspase-3 were significantly reduced by approximately 55% in the cortex and by 57% in the hippocampus of the RJ group compared to the AD model group (all *p* < 0.01), indicating that RJ can inhibit neuronal apoptosis.

### RJ Enhanced Anti-Oxidative Capacities in AD Rabbit Brains

The effects of RJ on anti-oxidative capacities of AD rabbit brains were measured. As shown in Figure 6, the SOD level was markedly reduced in the cortex and the hippocampus of the AD model group compared to the NC group (ANOVA with *post hoc* Tukey’s test, all *p* < 0.05). In contrast, the SOD level in the RJ group was significantly increased by approximately 27% in the cortex and by 13% in the hippocampus relative to the AD model group (all *p* < 0.05). On the other hand, the MDA content was increased in the cortex and the hippocampus of the AD model group compared to that of the NC group (ANOVA with *post hoc* Tukey’s test, all *p* < 0.01), and it was significantly reduced by approximately 18% in the cortex and by 22% in the hippocampus of the RJ group relative to the AD model group (all *p* < 0.05). In addition, the ROS/RNS levels were increased in the cortex and the hippocampus of the AD group compared to those of the NC group (ANOVA with *post hoc* Tukey’s test, all *p* < 0.05). The ROS level in the RJ group was dramatically decreased by 14.33% in the cortex and by 21.96% in the hippocampus relative to the AD model group (all *p* < 0.05). Moreover, the RNS level in the RJ group was also significantly decreased by 19.79% in the cortex and by 27.17% in the hippocampus relative to the AD group (all *p* < 0.05). These results suggest that RJ treatment can enhance anti-oxidative capacities in the AD rabbit brain.

### RJ Improved Neuronal Metabolic Activities in AD Rabbit Brains

We then used ^1^H-MRS to evaluate the changes of neruonal metabolic activities in rabbit brains, the results from which are presented in Figure 7. Compared with the NC group, the AD model group presented with decreased peaks of NAA and glutamate and increased peaks of Cho and mI, while RJ treatment reveresed these changes. Quantification analysis demonstrated that the levels of NAA/Cr and glutamate/Cr were significantly reduced in the brain of the AD model group when compared with the NC group (ANOVA with *post hoc* Tukey’s test, *p* < 0.05 and *p* < 0.01, respectively), while the levels of Cho/Cr and mI/Cr were significantly increased (ANOVA with *post hoc* Tukey’s test, *p* < 0.01 and *p* < 0.05, respectively). In specific, the levels of NAA/Cr and glutamate/Cr in the RJ group were increased by 18% and 25% respectively, relative to the AD model group (all *p* < 0.05). On the other hand, the levels of Cho/Cr and mI/Cr were markedly decreased by 16% and 26% respectively in the RJ group compared with the AD model group (all *p* < 0.05). These results indicate that RJ can elevate the levels of NAA and glutamate, and can reduce the levels of mI and Cho in the brain of AD rabbits with potential effects of improving neuronal metabolic activities in the AD brain.

## Discussion

Our study confirmed in a rabbit AD model that hypercholesterolemia promotes Aβ deposition and leads to neuronal loss, whereas RJ has the effects of reducing plasma TC and LDL-C levels, enhancing anti-oxidative capacities, ameliorating Aβ pathology and protecting neurons from damage. High cholesterol dietaries are known to promote Aβ generation in human brains, thereby increasing the risk of AD (Panza et al., 2006). Numerous studies have shown that rabbits fed with 1% or 2% cholesterol diet alone or plus trace amounts of copper in drinking water would develop AD pathology, including senile plaques, cognitive impairment and neuronal loss (Larry Sparks, 2004). In this study, we showed that rabbits receiving high cholesterol diets plus copper in drinking water resulted in elevated TC, LDL-C and Aβ (such as Aβ1–40 and Aβ1–42) levels, neuronal loss, and nuclear contraction or disappearance of Nissl bodies, confirming the crucial role of hypercholesterolemia in the development of AD as well as successfully establishing a rabbit AD model. In addition, we observed from an MRI analysis that there was an increase in ventricular volumes in rabbits following 12 weeks on a diet of 2% cholesterol plus copper in drinking water (data not shown), consistent with previous reports (Deci et al., 2012; Schreurs et al., 2013). Furthermore, we showed that RJ significantly reduced the levels of TC and LDL-C in AD rabbits, confirming the previously reported effects of RJ on lowering cholesterol and Aβ levels (Guo et al., 2007).

The effects of RJ on AD rabbits may be explained by its regulatory capabilities of proteins involved in Aβ production, transport, degradation and clearance, such as BACE1, RAGE, LRP1 and IDE. Regarding the regulatory role of RJ in Aβ production, BACE1 is a protease that initiates Aβ production and is mainly located on lipid rafts. Higher BACE1 protein levels were observed in the brain of AD patients (Fukumoto et al., 2002; Holsinger et al., 2002), while *BACE1* gene knocked-out mice were found not to produce Aβ (Vassar et al., 2014; Neumann et al., 2015). The activities of BACE1 are greatly influenced by the metabolism of cholesterol. The increase in Aβ levels caused by high cholesterol diets was found to associate with the up-regulation of BACE1 expression (Jaya Prasanthi et al., 2008). On the other hand, the reduction of cholesterol levels was found to inhibit the BACE1 enzyme activity and reduce Aβ production (Cui et al., 2011). In particular, cholesterol depletion has been shown to reduce the partitioning of APP into lipid rafts, precluding the interaction of BACE1 with lipid rafts and thus lowering Aβ production (Guardia-Laguarta et al., 2009). In this study, we found that BACE1 expression levels as well as cholesterol levels were significantly decreased by RJ, indicating a possible molecular mechanism underlying RJ-mediated decrease of amyloid plaques: RJ inhibits the APP cleavage of amyloid via lowering cholesterol levels and reducing the contact between BACE1 and lipid rafts.

The ability of RJ to reduce the Aβ deposition in the brain may also come from its ability to regulate the transport of Aβ through blood-brain barrier (BBB). Abnormal translocation of Aβ across BBB is considered to be a central link of AD pathogenesis. Recent studies have revealed RAGE and LRP1 as two key Aβ transporters on the BBB: RAGE mediates the transport of Aβ from the peripheral blood to the brain, whereas LRP1 mediates the transport of Aβ from the brain to the blood (Xi et al., 2013; Cirillo et al., 2015). In AD patients, the expression of LRP1 was down-regulated, while the expression of RAGE was up-regulated (Shibata et al., 2000; Deane et al., 2004), consistent with what we observed in AD rabbits. Furthermore, we found that RJ could increase the expression levels of LRP1 and inhibit the expression levels of RAGE in the brain of AD rabbits. This effect may be attributed to 10-hydroxy-trans-2-decanoic acid (HDEA), a compound in RJ that can pass through BBB. HDEA has similar effects as brain-derived neurotrophic factor (BDNF), and could potentially promote neurogenesis in mature brains (Hattori et al., 2007). These results suggest that RJ can regulate the translocation of Aβ via RAGE/LRP1 and therefore potentially reduce the Aβ deposition in the brain.

As for the degradation and clearance of Aβ, RJ plays a role in this as well and thereby further reduces the deposition of Aβ. IDE is one of the main enzymes for degrading Aβ in the brain. Increased amyloid plaque and Aβ levels, along with decreased IDE activity, were found in diet-induced insulin resistance APP transgenic mice (Farris et al., 2004). Meanwhile, increased levels of IDE can activate serine/threonine protein kinase B pathway, inhibit the activity of GSK-3β, and decrease abnormal phosphorylation levels of Tau protein (Wang et al., 2006). In addition, the clearance of IDE is related to the BBB transport, and it may contribute to the pathogenesis of AD by influencing insulin signal transduction (Del Campo et al., 2015). We found in this study that the expression levels of IDE were increased by RJ treatment, indicating yet another regulatory path for RJ to reduce Aβ levels.

Apart from Aβ pathology, AD may also benefit from the anti-oxidative effects of RJ. AD is associated with neuronal loss in the brains (Zou et al., 2015), of which apoptosis is a main factor. Oxidative stress was found to promote Aβ aggregation and cause damage to neurons through apoptotic pathways, whereas antioxidant enzymes and vitamin E could antagonize these effects (Mecocci et al., 1994). Known for its anti-oxidative and anti-aging effects, RJ contains rich antioxidant enzymes and vitamins, such as SOD (Min et al., 2004) and vitamin C. In this study, we examined the changes in apoptotic activities and oxidative stress in RJ treated rabbits using five markers: activated caspase-3, a protease involved in apoptosis that has been shown to participate in the pathological process of AD neuronal damage (Zhang et al., 2017); SOD, a free radical scavenging agent for evaluating free radical production; and MDA, which serves as a sensitive index for evaluating the oxidative stress response (Mancini et al., 2017); ROS/RNS play an important role in the survival and cell death signaling cascades that are essential for neurodegenerative disorders, and an increase in ROS/RNS levels (often associated with AD) will result in lipid and protein oxidation (Limongi and Baldelli, 2016). Studying the neurons in the cortex and the hippocampus of AD rabbits, we found that the number of neurons was significantly increased and that activated caspase-3 expression was markedly inhibited by RJ treatment. Moreover, the SOD level was significantly increased and the MDA content and ROS/RNS were decreased in the brain of AD rabbits after RJ intervention, indicating that RJ has the potential to prevent neuronal loss in AD by enhancing anti-oxidative capacities.

Our assessment of neuronal metabolic activities using ^1^H-MRS further revealed other potential mechanisms of how RJ may improve AD. First, we found that the levels of NAA and glutamate were notably increased in the brains of RJ-treated rabbits. NAA, a specific marker for neuronal viability and integrity (Chen et al., 2013), was reported to be at markedly reduced levels in AD patients (Moon et al., 2016). Glutamate, a major excitatory neurotransmitter that regulates learning, memory and movement, is a marker for neuronal survival and synaptic plasticity (Alvarez and Ruarte, 2004). The increased levels of NAA and glutamate found in RJ-treated rabbits indicate that RJ treatment can enhance neuronal metabolic activities in the brain of AD rabbits and may ultimately improve the cognitive abilities of AD. Furthermore, we found that RJ also increases the level of Cho and mI in the brain of AD rabbits. Cho, related to phospholipid metabolism of cell membranes, participates in the formation of cell membranes and myelin. Meanwhile, mI is a cell marker of glial since it is associated with the activation or proliferation of astrocytes (Zhang et al., 2014). In AD patients, Cho and mI levels were both reported to have increased due to the damage of brain cell membrane and the reactive proliferation of glial cells (Zhang et al., 2014). Our results confirmed that Cho and mI levels are increased in AD rabbits, and showed that these levels can be decreased by RJ treatment. This suggests that RJ treatment can potentially increase the stability of neural cell membrane and promote the activation of glial cells in the AD brain.

**Figure 8:**
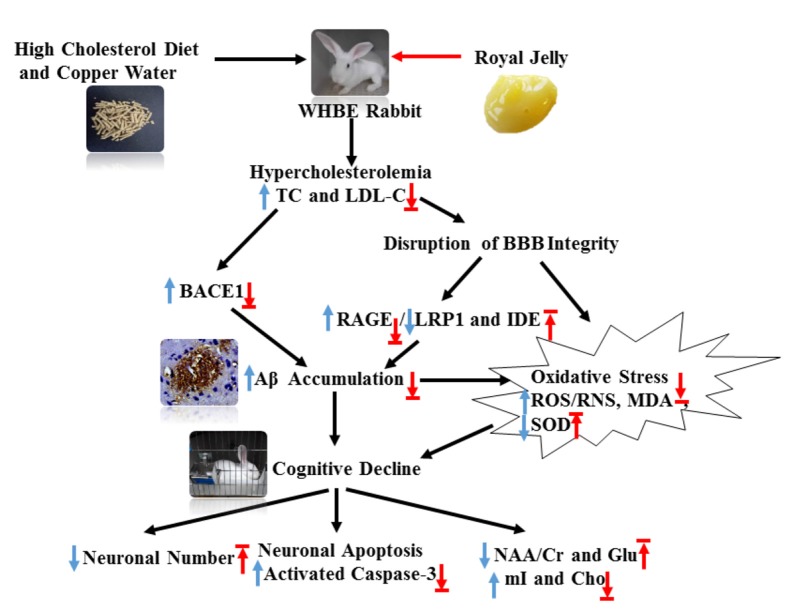
Schematic showing the possible mechanisms by which RJ improves hypercholesterolemia-induced rabbits with AD. AD conditions were induced by feeding rabbits with a high cholesterol diet plus copper in drinking water. RJ can reduce cholesterol levels, down-regulate BACE1 and RAGE expression, increase the expression levels of LRP1 and IDE, promote the degradation and clearance of Aβ, and finally reduce Aβ. Moreover, RJ also has anti-oxidative effects, and it enhances neuronal metabolic activities and prevents neuronal loss. TC, total cholesterol; LDL-C, low-density lipoprotein cholesterol; BACE1, β-site APP cleaving enzyme 1; BBB, Blood-brain barrier; RAGE, receptor for advanced glycation end products; LRP1, LDL receptor related protein 1; IDE, insulin-degrading enzyme; Aβ, amyloid β-protein; ROS, reactive oxygen species; RNS, reactive nitrogen species; MDA, malondialdehyde; SOD, superoxide dismutase; activated caspase-3, activated cysteinyl aspartate specific proteinases 3; NAA, N-acetylaspartata; Glu, glutamate; mI, myo-inositol; Cho, choline. Arrows pointing up or down indicate statistically significant increases or decreases (*p* < 0.05). Blue arrows indicate the changes in AD rabbits, while red arrows indicate the treatment effects of RJ.

In summary, this study showed that RJ can reduce cholesterol levels, down-regulate BACE1 and RAGE expression, increase the expression levels of LRP1 and IDE, promote the degradation and clearance of Aβ, and finally reduce Aβ (Figure 8). Moreover, we found that RJ has anti-oxidative effects and that it enhances neuronal metabolic activities and prevents neuronal loss. These results together suggest possible mechanisms by which RJ reduces Aβ deposition and prevents neuronal loss, providing preclinical evidence of the utility of RJ as a natural product for potentially preventing and treating AD.

## Author Contributions

FH, MC, YP designed the research project; YP, JX, CC, FC, PJ, MY and KZ performed the experiment; YP and MC analyzed the data; YP, CWH and FH wrote the article.

## Conflict of Interest Statement

The authors declare that the research was conducted in the absence of any commercial or financial relationships that could be construed as a potential conflict of interest.

## Footnotes

^1^http://www.njjcbio.com
